# Advances in genetic therapeutic strategies for Duchenne muscular dystrophy

**DOI:** 10.1113/EP085308

**Published:** 2015-08-04

**Authors:** Simon Guiraud, Huijia Chen, David T. Burns, Kay E. Davies

**Affiliations:** ^1^ Medical Research Council Functional Genomics Unit at the University of OxfordDepartment of Physiology, Anatomy and GeneticsOxfordOX1 3PTUK

## Abstract

**New Findings:**

- **What is the topic of this review?**
  This review highlights recent progress in genetically based therapies targeting the primary defect of Duchenne muscular dystrophy.
- **What advances does it highlight?**
  Over the last two decades, considerable progress has been made in understanding the mechanisms underlying Duchenne muscular dystrophy, leading to the development of genetic therapies. These include manipulation of the expression of the gene or related genes, the splicing of the gene and its translation, and replacement of the gene using viral approaches.

Duchenne muscular dystrophy is a lethal X‐linked disorder caused by mutations in the dystrophin gene. In the absence of the dystrophin protein, the link between the cytoskeleton and extracellular matrix is destroyed, and this severely compromises the strength, flexibility and stability of muscle fibres. The devastating consequence is progressive muscle wasting and premature death in Duchenne muscular dystrophy patients. There is currently no cure, and despite exhaustive palliative care, patients are restricted to a wheelchair by the age of 12 years and usually succumb to cardiac or respiratory complications in their late 20s. This review provides an update on the current genetically based therapies and clinical trials that target or compensate for the primary defect of this disease. These include dystrophin gene‐replacement strategies, genetic modification techniques to restore dystrophin expression, and modulation of the dystrophin homologue, utrophin, as a surrogate to re‐establish muscle function.

## Introduction

Duchenne muscular dystrophy (DMD) is a lethal X‐linked recessive disorder caused by the lack of the cytoskeletal protein dystrophin, which leads to progressive muscle wasting and weakness (Cohn & Campbell, 2000). Birth prevalence is estimated to be one in 5000 (Mendell *et al*. 2012), and *de novo* mutations continue to arise in all populations worldwide. Boys with DMD exhibit symptoms around 3–5 years of age, with abnormal gait, weakened proximal muscles and calf muscle pseudohypertrophy. The development of scoliosis coincides with wheelchair support by the age of 12 years (Emery, 1993) and patients usually die from respiratory and cardiac complications by 30 years of age (Bach *et al*. 1987). In Becker muscular dystrophy (BMD), partial deletion of dystrophin compromises its function, but patients can remain ambulant even at 60 years old (Emery, 1990). Despite exhaustive clinical attention for respiratory support, management of cardiac complications and corticosteroid treatment, there is currently no cure for this devastating disease. There is a great unmet clinical need because DMD is one of the most common genetic disorders, with an estimated 50,000 boys affected worldwide.

**Figure 1:**
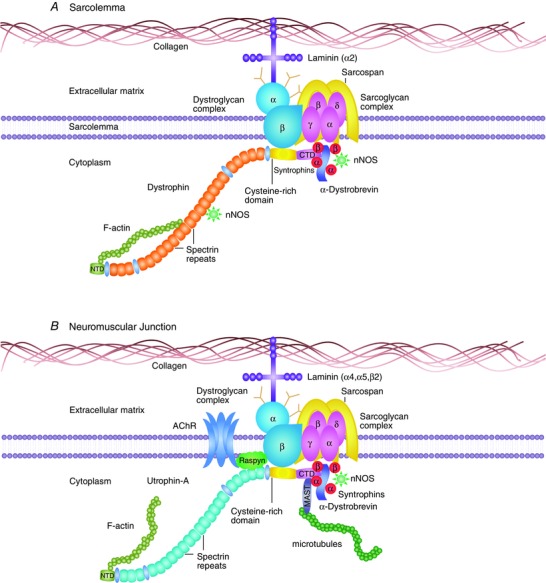
**The dystrophin and utrophin‐associated protein complexes** *A*, structure of the dystrophin‐associated protein complex (DAPC) at the muscle membrane. The DAPC acts as a link between myofibres and the extracellular matrix to provide stability at the sarcolemma. The central rod domain of dystrophin contains 24 spectrin repeats and four hinges. The N‐terminal domain (NTD) and specific spectrin repeats bind to cytosolic F‐actin to aid in shock absorbance that results from elastic recoil during muscle contraction or stretch. The cysteine‐rich domain (CRD) links dystrophin to the sarcolemmal‐bound β‐dystroglycan, which in turn binds to α‐dystroglycan to form the dystroglycan complex. This complex is further strengthened by binding to the sarcoglycans (α, β, δ and γ) and sarcospan at the sarcolemma as well as laminin α2 at the extracellular matrix. The C‐terminal domain (CTD) of dystrophin binds several cytosolic proteins, such as α‐dystrobrevin and syntrophins (α and β). These syntrophins can recruit neuronal nitric oxide synthase (nNOS) to the sarcolemma via their PDZ domains to regulate blood flow to the muscle. In addition, spectrin repeats 16/17 in dystrophin are also able to recruit nNOS. Dystrophin interacts indirectly with microtubules through ankyrin‐B and directly via spectrin repeats 20–23. Together, dystrophin and its associated proteins protect the sarcolemma from contraction‐induced injury. *B*, structure of the utrophin‐associated protein complex (UAPC) at the neuromuscular junction. The UAPCs have similar protective functions compared with the DAPCs, because utrophin shows 80% sequence homology to dystrophin. However, utrophin lacks the sequence corresponding to spectrin‐like repeats 15 and 19 of dystrophin and binds actin only through the NTD. Utrophin is unable to recruit nNOS directly via its spectrin repeats, although nNOS can still be recruited indirectly through the syntrophins. At the neuromuscular junction, UAPC also binds to Raspyn and is involved in the clustering of acetylcholine receptors (AChRs) to the membrane. In addition, the CTD of utrophin binds to Multiple asters (MAST), which associates with microtubules. The UAPC is linked to the extracellular matrix of the neuromuscular junction via laminins α4, α5 and β2.

Dystrophin is a 427 kDa protein expressed at the sarcolemma in skeletal muscle. The main function of dystrophin is to maintain the strength, flexibility and stability of muscle fibres (Davies & Nowak, 2006). Dystrophin provides an essential link between the dystrophin‐associated protein complex at the sarcolemma and the cytoskeleton (Fig. 1 *A*). In DMD, the pathological consequences of the lack of dystrophin and the loss of the dystrophin‐associated protein complex are dramatic, with a progressive cascade of events, such as continual influx of inflammation, repeated cycles of necrosis and regeneration, with satellite cell depletion, necrosis and fibrosis. The myofibres are more susceptible to contraction‐induced injury, which results in muscle wasting and premature death (Emery, 1990).

More than 25 years after the discovery of the dystrophin gene (Monaco *et al*. 1986), defining the molecular basis of the disease, the development of different DMD animal models, such as the *mdx* mouse (Bulfield *et al*. 1984) and the golden retriever muscular dystrophy dog (Sharp *et al*. 1992), has led to new understanding, delineating some key mechanisms of the pathology. Despite its mild phenotype, the *mdx* mouse is the most widely used DMD laboratory model. The *mdx* mouse has a stop codon in exon 23 of the gene and has been essential for the establishment of therapeutic approaches. Extensive preclinical studies in the *mdx* mouse assessing various approaches and their therapeutic efficacy have resulted in the first clinical trials (Table 1).

**Table 1:** Clinical trials using therapies targeting or compensating for the primary defect of Duchenne muscular dystrophy

					Percentage					
Drug		Mechanism		Delivery	of applicable	Current	Patients	Results to	Clinical trial	
name	Company	of action	Chemistry	route	patients	stage	involved	date	and/or URL	Reference(s)
Viral gene therapy										
Biostrophin	Asklepios Biopharmaceutica	Mini‐dystrophin	AAV	i.m.	100%	Phase 1 (completed)	6	Failed to establish long‐term dystrophin expression.	www.askbio.com	Mendell *et al*. (2010)
								Immune response against transgene		
Termination codon read through										
PTC‐124, Ataluren, Translarna	PTC Therapeutics	Nonsense mutation supression	Small molecule	Oral	10%	Phase 3	174	Slowed loss of walking ability in DMD patients at the lower doses tested	NCT01557400 www.ptcbio.com	Bushby *et al*. (2014)
Exon‐skipping										
Drisapersen	Prosensa Therapeutics	Exon skipping (exon 51)	2′OMePS oligonucleotide	s.c.	13%	Phase 2	186	Dystrophin restoration <20%	NCT01480245 www.prosensa.eu	van Deutekom *et al*. (2007)
								A 49 m difference in 6MWD (patients ≤7 years old).		
								Well tolerated. Reversible injection‐site reactions, renal event and subclinical proteinuria toxicity in kidney at high drug doses.		
Drisapersen	Prosensa Therapeutics	Exon skipping (exon 51)	2′OMePS oligonucleotide	s.c.	13%	Phase 3	53	Dystrophin restoration <20%.	NCT01153932 www.prosensa.eu	Voit *et al*. (2014)
								A 35 m difference in 6MWD (patients ≤7 years old) with continuous Drisapersen.		
								Well tolerated. Reversible injection‐site reactions, renal event and subclinical proteinuria toxicity in kidney at high drug doses.		
Eteplirsen	Sarepata Therapeutics	Exon skipping (exon 51)	PMO oligonucleotide	i.v.	13%	Phases 2/3	12	Dystrophin restoration <20%. Slower disease progression than natural history based on 6MWD. Continued stability of respiratory muscle function. Well tolerated.	NCT00844597 www.sareptatherapeutics. com	Kinali *et al*. (2009); Mendel *et al*. (2013)
Utrophin modulation										
SMT C1100	Summit Therapeutics	Utrophin modulation	Small molecule	Oral	100%	Phase 1	12	Well tolrated in healthy volunteers and in DMD patients.	NCT02383511 www.summitplc.com	Tinsley *et al*. (2011, 2015)
								Significant reduction in CK, AST and ALT levels was observed when compared with predose baseline levels.		

Abbreviations: 2′OMePS, 2′*O*‐methylphosphorothioate; 6MWD, 6 min walk distance test; AAV, adeno‐associated virus; ALT, Alanine aminotransferase; AST, Asparate aminotransferase; CK, Creatine kinase; DMD, Duchenne; PMO, phosphorodiamidate morpholino oligomer.

The major challenge for DMD is the need to develop a safe, systemic strategy to target all the muscles, including limb, respiratory and cardiac muscles, for all the patients. Here, we review the recent genetically based therapeutical advances and clinical trials that target and compensate the primary defect of the disease.

## Gene‐replacement strategies

Gene‐replacement approaches can treat all DMD patients, regardless of the mutation type. Approximately 20% of the wild‐type level of dystrophin is required to obtain a significant correction of the muscle pathology (Chamberlain, 1997). As the dystrophin gene (2.2 Mb) and the cDNA (11 kb) are exceptionally long, direct replacement of the dystrophin gene is challenging.

**Figure 2:**
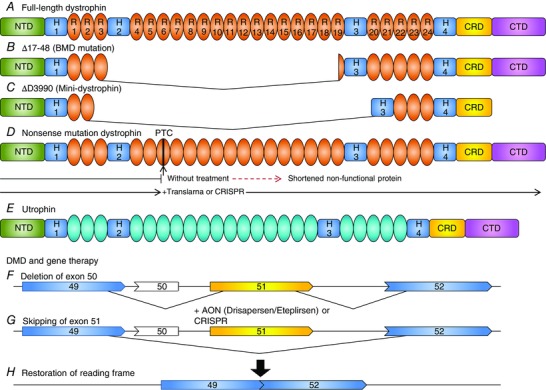
**Dystrophin and approaches to therapy** *A*, full‐length wild‐type dystrophin consists of an actin‐binding N‐terminal domain (NTD), hinge domains (H1–H4) and a cysteine‐rich domain (CRD) next to a carboxy‐terminal domain (CTD). Spectrin repeats (R1–R24) make up the rod domain. *B* and *C*, a mildly affected Becker muscular dystrophy (BMD) patient with exons 17–48 deleted, resulting in 46% of dystrophin deleted, has been reported (*B*) and forms the basis of mini‐dystrophin (*C*). *D*, in dystrophin containing a nonsense mutation causing a premature stop codon, Translarna allows read through or Clustered Regularly Interspaced Short Palindromic Repeats (CRISPR) can correct the mutation, restoring functional dystrophin. *E*, utrophin does not contain the same number of spectrin‐like repeats and can bind actin only through the NTD. Localization of nNOS to the sarcolemma is not possible with utrophin and some of the dystrophin mini‐genes, as observed for some mildly affected BMD patients. *F*, in DMD patients with a deletion of exon 50, exons 49 and 51 are out of frame. This leads to unstable pre‐mRNA, which is degraded without the protein being produced. *G* and *F*, using antisense oligonucleotides (such as Drisapersen or eteplirsen), skipping of exon 51 is promoted (*G*), resulting in restoration of the open reading frame (*H*).

Successful gene‐replacement therapy for DMD requires widespread and efficient delivery of the gene to all muscles. Adeno‐associated virus (AAV) vectors are suitable to achieve this goal but have a limited cloning capacity (4.6 kb), precluding delivery of the endogenous dystrophin gene. Based on a Becker patient with a very mild phenotype with 46% of dystrophin deleted (England *et al*. 1990), micro‐ and mini‐dystrophin have been delivered using AAV vectors (Harper *et al*. 2002) (Fig. 2B). In 2006, the first clinical gene therapy trial for DMD was conducted with six DMD boys with rAAV2.5.CMV.Δ3990 mini‐dystrophin (Fig. 2C). While transgene expression was undetectable, a T‐cell‐specific immune response to mini‐dystrophin was encountered in these patients (Mendell *et al*. 2010). Dystrophin‐specific T‐cells were detected after and before treatment, explaining the importance of T‐cell immunity to self and non‐self dystrophin epitopes. This emphasizes the necessity to prescreen patients for immunity to dystrophin before their enrolment in mini‐dystrophin gene therapy clinical trials. An immune response to the AAV was also reported. These adverse effects could be minimized by transient immunosuppression, as shown in the canine studies (Wang *et al*. 2007). Recently, a novel method for the delivery of the full‐length dystrophin DMD using a triple‐AAV trans‐splicing vector system was developed, which may result in better functional improvement (Koo *et al*. 2014).

Although more permanent in principle, dystrophin restoration by AAV delivery decreased significantly between 3–12 months post‐treatment, with important viral genome loss (Le Hir *et al*. 2013), explaining that optimal doses are required to induce substantial levels of dystrophin and maintain longer effects of the treatment. Many challenges remain in developing methods for systemic gene delivery and production of large volume titres of virus. Work is also in progress to improve viral transduction efficiency and reduce innate and acquired immune responses to allow repeated AAV–dystrophin delivery (Hayashita‐Kinoh *et al*. 2015).

## Gene product modifiers

### Exon skipping

The majority of DMD cases arise from partial dystrophin gene out‐of‐frame deletions (Bladen *et al*. 2015). These mutations disrupt the mRNA open reading frame and prevent the production of a functional dystrophin protein. Antisense‐mediated exon skipping uses modified and complementary RNA or DNA oligonucleotides (AONs) to modulate splicing of dystrophin pre‐mRNA, restore the reading frame and generate a BMD‐like truncated but partly functional dystrophin protein.

This promising strategy has been successfully induced in cells derived from the *mdx* mouse (Dunckley *et al*. 1998) and patient‐derived muscle cells (van Deutekom *et al*. 2001). Two proof‐of‐concept studies in DMD patients reported that a single direct intramuscular injection of the 2′*O*‐methyl‐ribo‐oligonucleoside‐phoshophorothioate Drisapersen (PRO051/GSK2402968; Prosensa/Biomarin; van Deutekom *et al*. 2007) or the phosphorodiamidate morpholino oligomers (PMOs) Eteplirsen (AVI‐4658; Sarepta Therapeutics; Kinali *et al*. 2009; Mendell *et al*. 2013) induced a specific skipping of exon 51 and produced variable amounts of dystrophin protein (Figs. 2F‐H). Despite promising initial results, Phase 2 trials with these two exon‐skipping strategies resulted in a variable increase of sarcolemmal dystrophin (<20%) and slower disease progression than natural history based on 6 min walk distance test (6MWD), a primary outcome measure in ambulatory DMD boys (Table 1). The most recent study with Drisapersen reported better results in the 6MWD test following continuous treatment with Drisapersen for 25 weeks (Voit *et al*. 2014). Several Phase 2/3 trials are currently in progress for Eteplirsen in ambulant and non‐ambulant DMD patients, and Sarepta Therapeutics could gain accelerated US Food and Drug Administration approval within the next year. The same may be true of Drisapersen for Prosensa/BioMarin.

In summary, the first generation of exon‐skipping strategies has been limited by low efficacy in cardiac muscle, poor cellular uptake, and rapid clearance from the circulation. Thus, repeated administrations are required to achieve therapeutic benefits, and the development of new chemistries and alternative delivery methods is essential.

Non‐spliceosome small nuclear RNA (snRNA), such as U7snRNA, induces efficient *in vitro* and *in vivo* exon‐skipping (Goyenvalle *et al*. 2004). Following a single injection of scAAV9‐U7ex23, near‐normal levels of dystrophin expression were achieved in all muscles examined, including the heart, and resulting in a remarkable rescue of the *mdx* muscle function. Nevertheless, this promising strategy faces immunological and delivery challenges specific to AAV vectors.

More recently, two emerging exon‐skipping strategies raised renewed hopes. The first is based on the use of cell‐penetrating peptides conjugated to uncharged antisense oligonucleotides in order to improve their potency and delivery. The early generation of peptide–PMOs were shown to correct the DMD phenotype and restore the muscle function without detectable toxicity or immune response but were weakly active in the heart. Recent reports suggest that a single low i.v. dose of highly active peptides named Pip (PMO internalizing peptides) in the *mdx* mouse results in high levels of dystrophin protein restoration, notably in cardiac muscle. Encouragingly, repeated peptide–AON treatments prevented the exercised induced progression of cardiomyopathy (Betts *et al*. 2015).

A new class of AONs made of tricyclo‐DNA (tcDNA) was recently described, with unique pharmacological properties and improved bio‐distribution, reaching skeletal muscle, heart and the brain (Goyenvalle *et al*. 2015). TcDNA was shown to be more effective than 2′*O*‐methyl‐ribo‐oligonucleoside‐phoshophorothioate or PMO in restoring dystrophin expression after the skipping of exon 23. In *mdx* mice, tcDNA induced widespread dystrophin recovery and improved the muscle function, with a normalization of the specific force, and revealed cardiac benefits. Interestingly, tcDNA–AON improves the respiratory function, benefits the CNS and shows a rescue of cognitive outcomes. Nevertheless, tolerability and safety of new exon‐skipping strategies, such as tcDNA and Pip–PMO, need to be assessed for regulatory toxicology, especially in the context of chronic treatment, before being tested in humans.

The limitation of exon‐skipping strategies is that they are intrinsically exon specific and therefore beneficial for only a subset of patients. Strategies that target exon 51 and exon 45 could be applicable to 13% and 8% of patients, respectively. Development of multi‐exon‐skipping strategies is theoretically possible. First proof‐of‐principle experiments were established with an AO ‘mixture approach’ for exon 45–55 skipping (Béroud *et al*. 2007). This strategy is theoretically applicable to 63% of DMD patients, but multi‐exon‐skipping shows high variability and low efficacy and is still at the preclinical stage.

### Suppression of premature stop codons

Approximately 10% of DMD patients have nonsense mutations (Bladen *et al*. 2015). Some antibiotics, such as gentamicin, promote translational read through of premature stop codons and increase dystrophin expression by up to 15% (Malik *et al*. 2010). Owing to the toxicity of gentamicin, a screening tool was developed to identify efficient compounds with appropriate safety profiles. Thus, Translarna (ataluren, PTC124) was identified as a first‐in‐class compound able to promote nonsense read through (Fig. 2D). An oral delivery of Translarna was well tolerated in humans after 48 weeks of treatment and resulted in a slower disease progression and a non‐significant 31 m improvement in a 6MWT (Bushby *et al*. 2014). No dystrophin expression data were presented. Currently, a Phase 3 trial is ongoing to determine the efficacy and safety of low doses of Translarna. Initially used to treat cystic fibrosis, Translarna lays the foundation to study other stop‐codon‐suppressing drugs in DMD.

### CRISPR/Cas9 system

 The bacterial Clustered Regularly Interspaced Short Palindromic Repeats (CRISPR)/CRISPR‐associated (Cas) system has emerged as a new promising and effective genome editing tool (Jinek *et al*. 2012). This system allows for sequence‐specific cleavage of target loci across the genome and has been successful in correcting the dystrophin mutation in the germ line of *mdx* mice (Long *et al*. 2014), in DMD patient myoblasts (Ousterout *et al*. 2015) and in induced Pluripotent Stem cells (iPS) (Li *et al*. 2015). However, successful transplantation of corrected myogenic cells remains an issue, and there may be a potential immunogenic response to the newly corrected gene product. In addition, off‐target effects of this new technology need to be evaluated.

## Utrophin modulation strategies

Utrophin is a promising candidate to compensate for the lack of dystrophin in all DMD patients independent of their mutation (reviewed by Guiraud *et al*. 2015a). Both utrophin and dystrophin have structurally similar N‐terminal, cysteine‐rich and C‐terminal domains (Love *et al*. 1989) and share many binding partners, such as β‐dystroglycan, α‐dystrobrevin‐1 and F‐actin (Ervasti, 2007). Utrophin and dystrophin have distinct expression patterns; utrophin is expressed in early developing muscles at the sarcolemma and is progressively replaced by dystrophin (Schofield *et al*. 1993). In adulthood, utrophin is expressed in a wide range of tissues; utrophin‐A localizes to the neuromuscular junction and myotendinous junction in muscles (Nguyen *et al*. 1991), while utrophin‐B is expressed in the endothelial cells (Weir *et al*. 2002). During muscle injury, utrophin‐A expression is switched back on at the sarcolemma in regenerated myofibres. In the absence of dystrophin, utrophin‐A expression is upregulated at the sarcolemma in the *mdx* mouse and DMD patients as part of the regeneration process (Helliwell *et al*. 1992).

Animal model studies have supported the use of utrophin modulation to correct the dystrophic phenotype. The transgenic *mdx* mouse *Fiona*, overexpressing utrophin three‐ to fourfold compared with wild‐type, did not develop muscular dystrophy (Tinsley *et al*. 1998). Importantly, in a broad range of murine tissues, no detrimental effects were noted with this ubiquitous overexpression of utrophin (Fisher *et al*. 2001). In addition, it was observed that early introduction of utrophin at birth prevented pathology, a result consistent with studies using dystrophin transgenes (Squire *et al*. 2002). Pharmacological interventions inducing a shift of fast‐twitch type II myofibres towards the slower, more oxidative type I myofibres also increase utrophin levels and confer a reduction of dystrophic phenotype in the *mdx* mouse (Chakkalakal *et al*. 2004). This functional benefit was completely negated in the double knock‐out *mdx* mouse, which is also deficient in utrophin, thus advocating utrophin as a crucial player in the mitigation of the dystrophic phenotype (Al‐Rewashdy *et al*. 2015). In dogs with golden retriever muscular dystrophy, less muscle fibrosis was observed when utrophin was delivered using intramuscular AAV injections (Cerletti *et al*. 2003).

Despite a strong functional redundancy for dystrophin, utrophin shows some distinct characteristics (Figs. 1B, 2E). Utrophin does not interact directly with microtubules (Belanto *et al*. 2014) or recruit neuronal nitrogen synthase (nNOS) to the sarcolemma to regulate blood flow to muscles (Li *et al*. 2010). Nevertheless, many BMD patients lacking the nNOS binding site in dystrophin remain mildly affected and ambulant without nNOS membrane localization, suggesting that there may be compensatory nNOS pathways (Li *et al*. 2010; Ramachandran *et al*. 2013). While some studies reported no relationship or a counterintuitive negative correlation of utrophin expression with BMD clinical severity (Vainzof *et al*. 1995; van den Bergen *et al*. 2014), the utrophin level is often complicated by the presence of mutant dystrophin and variable amounts of muscle regeneration. In DMD patients, a small increase in utrophin delays the age of wheelchair support (Kleopa *et al*. 2006), and utrophin can act as an effective surrogate for dystrophin in *mdx* muscles (Tinsley *et al*. 1998; Krag *et al*. 2004).

There are several ways to modulate utrophin levels, including direct protein replacement, stabilization of the protein/RNA and transcriptional upregulation of *utrn* RNA. Direct replacement of utrophin was achieved by delivery of the TAT protein transduction domain of the human immunodeficiency virus (HIV‐1) fused to recombinant full‐length utrophin (TAT‐Utr) and a shorter version (TAT‐μUtr). TAT‐μUtr yielded efficient biodistribution to a wide range of tissues and functional improvement of contractile strength in *mdx* mice (Sonnemann *et al*. 2009). Further investigation is warranted to assess the efficacy of this therapy in clinical trials. Ras homolog gene family, member A (RhoA), arginine butyrate and biglycan enhance the localization and stability of utrophin protein/RNA and hold great therapeutic promise (Fairclough *et al*. 2013).

The transcriptional upregulation of utrophin mRNA can be accomplished by activating utrophin promoter A, which is responsible for the expression of utrophin in skeletal muscle (Weir *et al*. 2002). Heregulin is capable of activating the N‐box, resulting in chromatin remodelling to increase utrophin levels (Krag *et al*. 2004). The non‐steroidal anti‐inflammatory drug Nabumetone is a positive regulator of promoter A, although its potential side‐effect of adverse cardiovascular events calls for more evaluation before it can be considered for DMD patients. Administration of 5‐amino‐4‐imidazolecarboxamide riboside increases peroxisome proliferator‐activated receptor γ coactivator1‐α, causing a fast‐to‐slow fibre‐type switch that is accompanied by an increase in utrophin (Hollinger *et al*. 2013). However, excessive fibre‐type switching may affect global muscle function. Strimpakos *et al*. (2014) delivered artificial zinc finger‐based transcriptional factors ‘Jazz’ and subsequently ‘UtroUp’ that bind exclusively to the utrophin promoter to mitigate the dystrophic phenotype in *mdx* mice.

Many of the above‐mentioned pharmacological agents face the challenge of systemic delivery without the use of invasive procedures in young DMD patients. Recently, in collaboration with Summit Therapeutics, we developed SMT C1100, an orally bioavailable drug that is able to modulate utrophin levels in the *mdx* mouse model and human DMD cells in a wide range of muscles, including the heart, diaphragm and skeletal muscles. Utrophin RNA and protein were increased twofold in skeletal muscles, and this was coupled with functional improvements in muscle fatigue testing and alleviation of muscle injury indicators (Tinsley *et al*. 2011). The Phase 1 clinical trial of SMT C1100 presented an excellent safety profile in healthy volunteers, and further trials are ongoing in DMD patients (Tinsley *et al*. 2015). Future clinical trials will seek to improve the plasma exposure of SMT C100 via dietary adjustments and test its efficacy in DMD patients. Investigation of compounds in the same chemical series as SMT C1100 has led to the identification of molecules with good bioavailability that also show efficacy in preventing the disease in the *mdx* mouse. Treatment with one such molecule, SMT022357, in the *mdx* mouse showed an increase in utrophin expression in skeletal, respiratory and cardiac muscles, resulting in improved sarcolemmal stability and reduction of regeneration, necrosis and fibrosis. All these improvements combine to protect the *mdx* muscle from contraction‐induced damage and enhance physiological function (Guiraud *et al*. 2015 *b*). In summary, these studies show proof of principle that modulation of utrophin expression is a viable therapeutic approach. Clinical trials with SMT C1100, optimization of further compounds in this chemical series and the discovery of other chemical entities with similar utrophin‐modulating effects may eventually lead to an effective disease‐modifying therapy for DMD regardless of the dystrophin mutation.

## Final perspectives

The identification of the dystrophin gene has led to an explosion in our understanding of DMD and the definition of rational therapeutic approaches through extensive preclinical studies. Early encouraging clinical trials bring hope that effective treatment for DMD is now possible.

## Additional information

### Competing interests

K.E.D. is on the Scientific Advisory Board of Prosensa plc and Summit Therapeutics. K.E.D. is a shareholder of Summit Therapeutics.

### Funding

K.E.D. and S.G. are funded by the Medical Research Council, D.T.B. is funded by Muscular Dystrophy UK, and H.C. is funded by Summit Therapeutics. This work is also supported by the Muscular Dystrophy Association USA.
